# Mapping the Silent Threat: A Comprehensive Analysis of Chagas Disease Occurrence in Riverside Communities in the Western Amazon

**DOI:** 10.3390/pathogens13020176

**Published:** 2024-02-15

**Authors:** Daniela da Silva Paixão, Fernanda Portela Madeira, Adila Costa de Jesus, Hêmilly Caroline da Silva Paixão, Juliana de Souza Almeida Aranha Camargo, Mariane Albuquerque Lima Ribeiro, Leandro José Ramos, Jader de Oliveira, João Aristeu da Rosa, Paulo Sérgio Bernarde, Antonieta Pereira Relvas, Sergio de Almeida Basano, Luis Marcelo Aranha Camargo, Dionatas Ulises de Oliveira Meneguetti

**Affiliations:** 1 Postgraduate Program in Health Sciences in the Western Amazon, Federal University of Acre, Rio Branco 69.920-900, Brazil; dani.paixao13@hotmail.com (D.d.S.P.); fernanda.madeira@ufac.br (F.P.M.); adila.jesus@ufac.br (A.C.d.J.); mariane.ribeiro@ufac.br (M.A.L.R.); snakebernarde@hotmail.com (P.S.B.); dionatas.meneguetti@ufac.br (D.U.d.O.M.); 2 Multidisciplinary Center, Federal University of Acre, Cruzeiro do Sul 69.980-000, Brazil; 3 Postgraduate Program in Science, Innovation and Technology for the Amazon, Federal University of Acre, Rio Branco 69.920-900, Brazil; hemillycspaixao@gmail.com; 4 Institute of Biomedical Sciences, University of São Paulo, Monte Negro 05.508-000, Brazil; lycosa@gmail.com (J.d.S.A.A.C.); basanosergio22@gmail.com (S.d.A.B.); spider@usp.br (L.M.A.C.); 5 Center for Health and Sports Sciences, Federal University of Acre, Rio Branco 69.920-900, Brazil; leandro.ramos@ufac.br; 6 Public Health Entomology Laboratory, Department of Epidemiology, Faculty of Public Health, University of São Paulo, São Paulo 01.246-904, Brazil; 7 Faculty of Pharmaceutical Sciences, Paulista State University “Júlio de Mesquita Filho”, Araraquara 14.800-700, Brazil; joao.aristeu@unesp.br; 8 Municipal Health Department of Humaitá, Humaitá 69.800-000, Brazil; antonietarp@hotmail.com; 9 National Institute of Epidemiology of the Western Amazon, Tropical Medicine Research Center, Porto Velho 76.812-329, Brazil; 10 Application College, Federal University of Acre, Rio Branco 69.920-900, Brazil

**Keywords:** vectors, American Trypanosomiasis, *Trypanosoma cruzi* and Amazônia

## Abstract

Chagas disease (CD) is a typical tropical illness caused by *Trypanosoma cruzi*. The objective of this study was to assess the prevalence of Chagas disease in communities in two states of the Brazilian Amazon. Data collection occurred in July in the Alto Juruá region of Acre and in December in the communities of Humaitá, Amazonas, in 2019. A total of 477 participants were included in the study. In the communities of Alto Juruá, triatomine collections and analyses of *T. cruzi* infection were also carried out. All confirmed cases were found in the state of Acre, resulting in a total prevalence of 1.67. Of these eight cases, seven underwent ECG, all of which were concluded as normal by the physician team’s cardiologists. Seventeen triatomine bugs, all belonging to the *Rhodnius* genus, were captured. The natural infection rate by *T. cruzi* was 25% in the Nova Cintra community and 66.67% in the Boca do Moa community (Alto Juruá). This research found that more than 1% of the studied population exhibited positive serological results for Chagas disease in the riverine communities during the study period, representing a small portion of cases among those who have not yet been diagnosed.

## 1. Introduction

Chagas disease (CD) is categorized as a neglected tropical disease, with an estimated 8 million people infected with *T. cruzi* worldwide and over 10,000 deaths annually (WHO, 2019) [1]. This illness primarily affects countries in Central and South America, which are considered endemic regions (Tanowitz, Weiss, Montgomery, 2011) [2]. In recent years, this pattern has been undergoing some changes due to emigration (mainly of Latin Americans), resulting in cases being reported in non-endemic countries such as Canada, the United States, European countries, and some Asian countries (Schmunis, 2007; Imai et al., 2014; Garcia et al., 2015; Méndez et al., 2015; Fidaldo et al., 2018) [3,4,5,6,7].

In Latin America, the countries with the highest rates of Chagas disease cases are Argentina, Bolivia, and Brazil (Coura, Borges-Pereira, 2010; Arnal, 2019) [8,9]. In Brazil, over a thousand cases of acute Chagas disease were confirmed between 2012 and 2016, resulting in an average annual incidence of 0.1 cases per 100,000 inhabitants. The Brazilian Amazon accounted for over 96% of these reported cases (Brasil, 2019) [10].

Recent studies have confirmed the presence of *T. cruzi*-positive triatomine bugs in remote riverine communities, away from major urban centers. This finding is significant for conducting research involving populations residing in these areas, as it increases the potential for these communities to become outbreak settings for Chagas disease (Madeira et al., 2020; Jesus et al., 2021; Souza et al., 2021) [11,12,13].

In addition to the presence of *T. cruzi* in vectors, other factors that need to be considered in studies in this region are the environmental and socioeconomic characteristics of the population. These include the predominant type of construction materials used in houses and their locations, as the transmission of Chagas disease to humans can be dependent on the proximity of cohabitation between vector and host or direct contact with *T. cruzi* (Kruse et al., 2019) [14]. Factors such as extractive practices, where the routine consumption of locally extracted and homemade food without proper preparation occurs, also play an important role (Kruse et al., 2019) [14]. 

Therefore, studying the occurrence of Chagas disease in the population of these communities is necessary to contribute to the early diagnosis of new cases and disease prevention. Thus, the present study aimed to investigate the occurrence and prevalence of Chagas disease in communities in two states of the Western Amazon region of Brazil, Acre and Amazonas. 

## 2. Materials and Methods

### 2.1. Study Areas

This study was conducted in communities from three municipalities: two in the state of Acre (Cruzeiro do Sul and Rodrigues Alves) and one in Amazonas (Humaitá), all located in the Brazilian Amazon region.

The state of Acre comprises twenty-two municipalities, including Cruzeiro do Sul and Rodrigues Alves, located in the Vale do Juruá region. According to the latest demographic census conducted in Brazil in 2010, the rural population of Cruzeiro do Sul represented nearly 30% of its total population, while in Rodrigues Alves, the situation was the opposite, with approximately 70% of its population residing in rural areas (Acre, 2017) [15].

In each municipality, one community was selected for this study. The Boca do Moa community in Cruzeiro do Sul and the Nova Cintra community in Rodrigues Alves were chosen. Nova Cintra is a relatively understudied area situated in one of the main parasite circulation zones. The presence of *T. cruzi*-positive triatomine bugs was confirmed in both communities, along with positive cases of Chagas disease in Nova Cintra in 2016 (Muniz, 2016; Madeira et al., 2020; Jesus et al., 2021) [11,12,16].

The Boca do Moa community is located approximately three kilometers from the urban perimeter of Cruzeiro do Sul municipality. It comprises around 90 families residing in houses predominantly built on stilts. During the summer, access to the community is possible by both land and river routes, while during the winter, only river access is available, as the dirt road becomes impassable. The primary economic activities in the community include fishing, cassava cultivation, and subsistence farming. Additionally, residents engage in the extraction of *Euterpe* spp. (açaí), *Mauritia flexuosa* (buriti), and *Oenocarpus bacaba* (bacaba) (Jesus et al., 2021) [12].

Nova Cintra is situated in the rural area of Rodrigues Alves municipality, approximately 12 km away from the urban area. It is home to over 58 resident families, with access primarily through an unpaved road (Madeira et al., 2020) [11]. The socioeconomic profile is similar to that of Boca do Moa.

**Figure 1:**
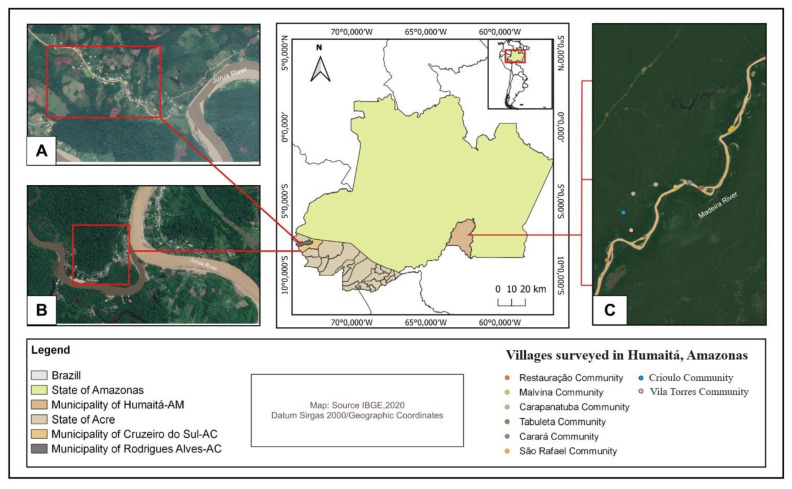
Map of the study areas with the following descriptions: (**A**) Nova Cintra Community in the municipality of Cruzeiro do Sul, Acre, Brazil; (**B**) Boca do Moa Community in the municipality of Rodrigues Alves, Acre, Brazil; (**C**) Humaitá Communities, Amazonas, Brazil.

The state of Amazonas has 62 municipalities, including Humaitá, located in the southern part of the state on the right bank of the Madeira River, about 675 km away from Manaus. It shares borders with the municipalities of Tapauá and Canutama to the west; Manicoré to the north, east, and west; and the state of Rondônia to the south, approximately 200 km from the capital, Porto Velho (IBGE, 2017) [17]. The municipality has an estimated population of 53,383 inhabitants and a territorial area of 33,111 km^2^. The region experiences a rainy tropical climate from October to March, with a dry period from June to August, including transitional periods (Pedreira Junior et al., 2018) [18]. Along the river, riverine communities were selected for this study, as they are served by the Fluvial Primary Healthcare Unit (UBS Fluvial). The communities included in this research are Carará, Crioulo, Boca de Carapanatuba, Tabuleta, Vila Torres, Malvina, São Rafael, and Restauração, which are between 40 and 120 km away from the city of Humaitá. Access to these communities is by river along the Madeira River. The location of the study areas can be seen in Figure 1.

### 2.2. Characteristics of the Studied Population

Prior contact was made with local health agents and community leaders, who informed the local population about the services to be provided, the location, and the date of the activity. All individuals seeking assistance were attended to, reaching over 70% of the populations in the communities. The service began with an explanation of the consent form, followed by the collection of information from participating residents, starting with place of residence, gender, and age. 

Following the anamnesis, a physical examination was conducted, measuring blood pressure, body temperature, and body weight. The following cases were referred to the medical team for a more detailed physical examination and possible follow-up: individuals with fever, those who had contact with the triatomine bug, chronic cases of Chagas disease, individuals who had previous Chagas disease, those who presented any symptoms of the acute phase of Chagas disease in the last three weeks or currently, and people who live or are related to Chagas disease carriers. Additionally, medical and nursing care was provided to the general population as needed. When it was determined during the screening that an electrocardiogram (ECG) was necessary, the research participants were directed to a private room for the examination.

### 2.3. Prevalence of Chagas Disease

For each participant, 10 mL of blood was collected through venous puncture using disposable needles and syringes. Slides for blood smears (to search for acute cases) were prepared at the time of puncture, using 10 to 20 µL of blood and the thick smear method stained with Giemsa. Once properly identified, the slides were placed in slide boxes at room temperature (Lima, 2019) [19].

In the communities of Acre, for serological testing, the collected material was sent to the laboratory at the Federal University of Acre (UFAC) campus Floresta in Cruzeiro do Sul. There, it was processed and centrifuged at 3000 rpm for 10 min to obtain a 2 to 4 mL fraction of serum, which was then forwarded to the Central Public Health Laboratory of Acre (LACEN).

In the communities of Humaitá, processing was carried out at the UBSF laboratory, and the samples were subsequently sent to the Central Public Health Laboratory of Rondônia (LACEN). In both locations, serology was performed using an Enzyme-Linked Immunosorbent Assay (ELISA) as a screening method. A commercial Biolisa Chagas Recombinant Kit from Bioclin, provided by the Ministry of Health of Brazil, was used. This kit includes recombinant *T. cruzi* antigens.

Positive cases, and those required to establish the “diagnostic standard” with two methodologies, were also sent to the Ezequiel Dias Foundation (FUNED) and the Octávio Magalhães Institute in Minas Gerais (which is one of the reference laboratories in Brazil), where the Indirect Hemagglutination (HAI) test was conducted. In two cases, a third serological method, Indirect Immunofluorescence (IFI) for IgM and IgG, was applied, as the previous results were discordant (Meis, Castro, 2017; Lima, 2019; PAHO, 2019) [19,20,21].

**Figure 2:**
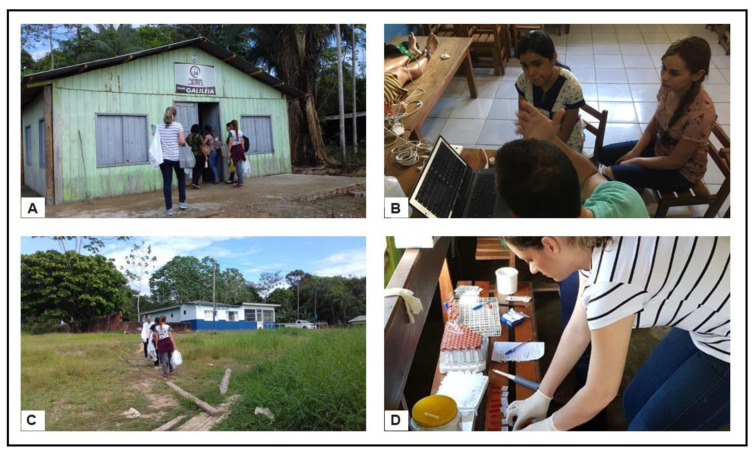
Collections in the Boca do Moa community, Cruzeiro do Sul, Acre, Brazil. (**A**) First day of collections at a local church; (**B**) realization of ECG; (**C**) second day of collections carried out at a daycare center; (**D**) preparation of slides and material for serology.

The collections were carried out in the municipality of Cruzeiro do Sul on 12–13 July 2019 in the Boca do Moa community. On the first day, after organizing the space, the collection of biological material for hemocytology, serology, and PCR for Chagas disease began at 10 a.m. at the local evangelical church. On the second day, the work started at 7 a.m., this time at a daycare center next to the evangelical church. Electrocardiograms (ECGs) were performed in a private space, and more patients were examined with blood collection (Figure 2).

The second community studied was Nova Cintra in the municipality of Rodrigues Alves. On 15 July 2019, the work began at 10 a.m. at the Community Basic Health Unit. The activities carried out included screening, ECG, health education, and the collection of blood samples from patients examined for hemocytology, serology, and PCR for Chagas disease. On the following day, more patients were attended to at the same location.

**Figure 3:**
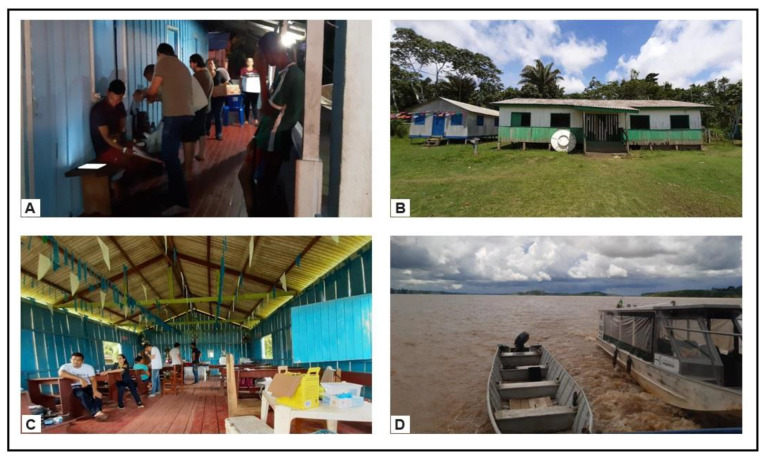
Collection in riverside communities of Humaitá, Amazonas, Brazil. (**A**) House in the Tabuleta community; (**B**) school in the Carará community; (**C**) church in the Creole community; (**D**) boats used to visit other communities.

In Humaitá, eight riverside communities along the Madeira River were studied, reached by a Basic River Health Unit from 9 December 2019 to 13 December 2019, totaling five days of work. The first community studied was Tabuleta, where the vessel stayed for one day and one night. The following day, the UBS proceeded to the Carará community, as shown in Figure 3. Subsequently, it visited the Crioulo community, and the remaining communities were reached using a boat with an outboard engine, as depicted in Figure 3.

### 2.4. Collection of Triatomines and Analysis of T. cruzi Infection

The collections were conducted in the Boca do Moa and Nova Cintra communities on 18 and 19 October 2019. An active search for triatomines in peridomicile and palms was conducted in the Alto Juruá communities, specifically in Boca do Moa and Nova Cintra, where positive cases of Chagas disease were identified. 

The peri-domiciliary search involved a meticulous examination for triatomines in areas displaying characteristics of potential shelters for these insects. The search took place around residences in debris such as woodpiles; bricks; construction materials; and structures housing animals, such as chicken coops.

In each community, two Attalea butyraceae trees were examined. These trees were selected for having a voluminous canopy and were cut down using a chainsaw. Subsequently, the bracts (where a large quantity of invertebrates and small vertebrates can inhabit) were removed one by one to facilitate the capture of triatomines (Meneguetti et al., 2012) [22].

The collected triatomines were stored in containers with perforated lids and kept humid using accordion-folded cardboard to retain the insects’ feces and prevent damage to the material (Obara, Wanderley, Silva, 2014) [23]. Each collected specimen was labeled with information about the location and date of collection and sent to the Tropical Medicine Laboratory at the Federal University of Acre for the identification and analysis of *T. cruzi* infection based on the external morphological characteristics described in the dichotomous key by Lent and Wygodzinsky (1979) [24] and other recent references (Rosa et al., 2012; Galvão et al., 2014; Rosa et al., 2014) [25,26,27].

Triatomine infection was analyzed using optical microscopy. The digestive content of the insect was diluted in a 0.9% saline solution on slides and observed under 1000× magnification, both fresh and stained with Panótico Rápido^®^ (triarylmethane 0.1%, xanthene 0.1%, and thiazine 0.1%). When positive, a molecular analysis was performed according to a method reported by Fernandes et al. [28]. This method amplifies a portion of the non-transcribed spacer of the mini-exon gene that is different between *T. cruzi* and *T. rangeli* species and between *T. cruzi* strains. The generated fragments vary in length between 100 and 250 base pairs. The oligonucleotide sequences used were TC1: (5-ACACTTTCTGGCGCTGATCG-3); TC2: 250 bp, (5-TTGCTCGCACACTCGGCTGCAT-3); Z3: 150 bp, (5-CCGCGCACAACCCCTATAAAATG-3); TR: 100 bp, (5-CCTATTGTGCCATCTTCG-3) and EXON: (5-TACCAATAGTACACACAACTG-3′)9.

The reaction mixture consisted of 100 pmol of each primer and 150 μM deoxynucleotide triphosphates in a buffer consisting of 10 mM Tris-HCl (pH 8.3), 1.5 mM MgCl_2_, 25 mM KCl, 0.1 mg/mL bovine serum albumin, 2.5 U TaqDNA polymerase, and approximately 10 ng of the genomic DNA sample, totaling a final volume of 50 μL with Type 1 water.

The thermal cycling conditions during each step were as follows: an initial step of 5 min at 95 °C; 34 cycles of 30 s at 94 °C, 30 s at 55 °C, and 30 s at 72 °C; and a final extension of 10 min at 72 °C. The following reference strains were used as controls in each reaction: TC1 X10 Clone 1, TC2 strain Y, Z3 Esmeraldo Clone 1, and *T. rangeli* R1625. The amplified products were electrophoresed on a 2% agarose gel at 100 V for 1 h. After electrophoresis, the DNA was stained with ethidium bromide and visualized under ultraviolet light. A molecular marker of 50 base pairs was used as a size control for the amplified fragments.

The Chi-square test (using EpiInfo 7 software) was applied to compare the positive cases of Chagas disease among the studied communities. The same test was also used to compare the infection of triatomines with *T. cruzi* according to the developmental stage of the triatomines. This study was submitted to Brazil Platform and approved by the Research Ethics Committee involving Human Subjects (CEP) at the Institute of Biomedical Sciences, University of São Paulo—ICB/USP, under the following approval number: 4.026.022. The collection of triatomines was conducted with authorization from the Brazilian Institute of Environment and Renewable Natural Resources, license no. 52260-1.

## 3. Results

The study population from the riverside communities was mostly female, although with a slight difference. Similarly, in terms of age, the majority of the participants were 18 years or older (Table 1).

The communities with the highest number of participants were Nova Cintra, Carará, and Boca do Moa, while the communities with the fewest participants were São Rafael, Malvinas, and Restauração.

### 3.1. Prevalence of Chagas Disease

There were no positive acute cases identified through the thick smear method; cases were only detected through serology. The prevalence of positive cases was determined for each studied municipality based on the sample size (Table 2).

The highest prevalence of positive cases was found in the municipality of Rodrigues Alves in the community of Nova Cintra. The other location with a positive case was in Cruzeiro do Sul, in the community of Boa do Moa. When comparing the data from the two communities (Nova Cintra and Boca do Moa), both in the Alto Juruá region, the state of Acre, with those from the communities of Humaitá (where no cases were found), a statistical significance of *p* < 0.01 could be observed. However, when comparing the communities of Nova Cintra and Boca do Moa, no statistical difference in positive cases was observed, with *p* > 0.05. The eight positive cases of Chagas disease are detailed in Table 3, showing age, gender, and the diagnostic tests that were realized.

It was found that the age ranged from 16 to 63 years, with 50% composed of men and 50% of women. There were also two indeterminate results, with three diagnostic methods having been employed in these cases.

### 3.2. Electrocardiograms

With the aim of identifying possible cardiac alterations in people with DC, after a medical history assessment, electrocardiograms (ECGs) were conducted exclusively in the Alto Juruá region. In the community of Boca do Moa, 11 ECGs were performed, while in the Nova Cintra community, 8 ECGs were conducted. Only one child, an 8-year-old female with potential rheumatic fever (audible aortic and tricuspid ejection murmurs and ECG alterations in ventricular repolarization between V1 and V3), showed abnormalities, without ongoing monitoring and without a confirmed case of DC.

**Figure 4:**
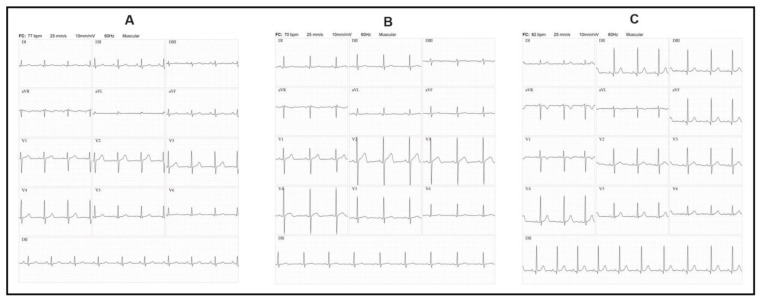
Electrocardiogram of three patients with chronic Chagas disease. (**A**) Female patient, 50 years old; (**B**) female patient, 16 years old; (**C**) female patient, 30 years old.

Patients with a history of Chagas disease in 2014 (one case) and 2016 (six cases) were all re-examined. The ECG results were sent for analysis to cardiologists at the Heart Institute of the Hospital das Clínicas of the Faculty of Medicine of the University of São Paulo (INCOR–FMUSP). Among the seven cases, only one had a right bundle branch block in the heart. All patients were afebrile and had normal vital parameters. Only two reported occasional palpitations (Figure 4).

### 3.3. Triatomines and Analysis of T. cruzi Infection

In the active search in the peridomicile, no triatomines were found. However, in the search in palms, seventeen triatomines were captured—eight in the Nova Cintra community (Table 4) and nine in the Boca do Moa community (Table 5)—with infection rates of 25% and 66.67% for *T. cruzi*, respectively.

It is also important to highlight that, during the collection, the residents of the Boa do Moa community themselves handed over an adult triatomine of the species *R. montenegrensis*, which was collected inside the community’s church. This demonstrates the occurrence of this species in human constructions; however, these data were not included in the current analysis.

When comparing the developmental stages of the triatomines, no significant differences were observed in any of the communities, with *p* > 0.05.

## 4. Discussion

The states of Acre and Amazonas have infection rates with frequencies above the national average, although they are lower than those of the state of Pará, which has the highest number of Chagas disease cases in Brazil. Out of the total sample of 477 individuals examined using serological methods, eight cases of chronic Chagas disease were diagnosed, resulting in a total prevalence of 1.67 (eight cases) for the studied population in this research. This prevalence was 1.44 (one case) in BC and 3.58 (seven cases) in NC. However, there were no positive results for Chagas disease in the Humaitá communities. This significant difference might be related to the occurrence of Chagas disease outbreaks in the Alto Juruá region, as seen in the case of Nova Cintra, where outbreaks had previously occurred through oral contamination via the consumption of an açaí beverage by a family group in 2016 (Carvalho, 2016; Muniz, 2016; Oliveira et al., 2018) [16,29,30]. The data are also consistent with those of a study on Chagas disease cases over a 10-year period in the state of Amazonas, where no positive cases were recorded in the municipality of Humaitá (Menezes et al., 2019) [31].

According to data from the National Notifiable Diseases Information System (SINAN) of the Ministry of Health since 2005, the epidemiological profile of Chagas disease has shown an increasing trend in oral transmission, surpassing vector transmission. Outbreaks involving people consuming the same contaminated beverage or food, particularly after the harvest of açaí and bacaba, are common (Oliveira et al., 2018; Brasil, 2020) [30,32]. This aligns with the increasing trend in oral transmission cases in the north region, where 150 outbreaks were reported over a 9-year period (Brasil, 2020; Santos et al., 2020) [32,33].

No acute cases were recorded in this study, which could have been detected in the initial phase using the thick smear diagnostic method. This might be attributed to the data collection timing, which was around mid-July in Alto Juruá and early December in Humaitá. A study on the northern region between 2007 and 2018 observed a higher incidence of Chagas disease notifications between December and April (Madeira et al., 2020) [11]. However, this seasonality can vary based on registered and potential outbreaks (Oliveira et al., 2018; Menezes et al., 2019) [30,31].

In this research, the age range of positive cases was between 16 and 63 years, which is in line with the literature, where Chagas disease cases are concentrated between 20 and 59 years. Of the eight cases in this study, five were within this age range. Regarding gender, there were equal occurrences for both sexes, showing that the risk is the same for both. This is similar to some studies where, even though males were more affected, at 53.56% (Alencar et al., 2020; Brasil, 2020; Brasil, 2021) [32,34,35] and 59.5% (Oliveira et al., 2018) [30], there was no significant difference (*p* > 0.05) between the genders.

Of the eight cases of Chagas disease, all in the chronic phase, seven underwent ECG testing, and all were concluded to have a normal ECG result according to analyses by cardiologists from INCOR–FMUSP. Cardiac involvement in the chronic phase of the disease is a significant factor in morbidity and mortality. In endemic countries, it represents one of the main causes of non-ischemic cardiomyopathy, heart failure, and sudden death (Lima et al., 2021) [36]. In a study of stable chronic Chagas disease patients suspected of having Chagas microvascular coronary disease, just over 33% had normal ECG results, concluding that alterations in this exam are frequent when patients have cardiac complications (Campos et al., 2020) [37], which were absent in the patients studied in this research.

Seventeen triatomines were captured, all belonging to the *Rhodnius* genus, which is the most commonly found species in palm trees in the Amazon region (Meneguetti et al., 2012) [22]. These data align with those of other studies conducted in the Alto Juruá region, where the most prevalent genus in palm trees was *Rhodnius* (Madeira et al., 2020; Moraes et al., 2020; Jesus et al., 2021) [11,12,38].

Palm trees of the Attalea genus are commonly found in this region, constituting another alert factor for the local population (Calderón et al., 2020) [39]. A recent study involving this palm tree detected *Rhodnius* infestation in 18 out of 280 palms. Deforestation was identified as a facilitator for Chagas disease vectors, indicating an elevated risk of vector contamination due to the proximity of the vectors to the population (Santos et al., 2021) [40]. Another important factor is that, in some situations, palm leaves are still used to cover dwellings, which could potentially lead to the domiciliation of these triatomines. This has been observed in some South American countries (Cuba et al., 2002; Matias et al., 2003) [41,42], indicating a high potential risk for vector-borne contamination due to the proximity of the vector to the population (Monsalve-Lara et al., 2021) [43].

The natural infection rate with *T. cruzi* was 25% in the Nova Cintra community and 66.67% in the Boca do Moa community, similar to other studies conducted in the Amazon region (Massaro et al., 2008; Meneguetti et al., 2012; Madeira et al., 2020; Moraes et al., 2020; Jesus et al., 2021) [11,12,22,38,44]. In this study, no significant difference in the infection percentage between the developmental stages of the triatomines and *T. cruzi* infection was observed. This is unlike the findings of studies by Meneguetti et al. (2012) [22], Moraes et al. (2020) [38], and Prati et al. (2020) [45], where a higher infection rate with trypanosomatids was found in closer-to-adult developmental stages. This difference might be due to the sample size in this study being smaller than that in the cited studies.

According to Alencar et al. (2020) [34], currently, the Amazon region accounts for over 95% of Chagas disease cases in Brazil. This number might still be underestimated due to the scarcity of symptoms in the acute phase of the disease, which often presents as a nonspecific febrile illness and can be confused with other endemic diseases in the northern region, such as malaria. When not identified in the acute phase, the transition from indeterminate to clinical forms of the disease is delayed and might not even be identified. Another challenge in this region is the difficult access to remote locations. Despite having the majority of cases in Brazil, the northern region ranked third with 33.7% of the total requests for laboratory tests for Chagas disease diagnosis in the last three years, according to the 2020 epidemiological report (Schmidt, Marin-Neto, 2020; Brasil, 2021) [35,46].

## 5. Conclusions

It was observed that over 1% of the studied population in the riverside communities tested positive for Chagas disease serology during the period of this research. This represents a small portion of cases among those who have not yet been diagnosed, given that current outbreaks are directly associated with acute Chagas disease infections, and no outbreaks occurred in these locations during the study period. Among the research participants, no acute cases were diagnosed, only chronic and asymptomatic cases. This implies that some cases may not have been identified by patients who typically seek medical attention only when presenting symptoms. This further suggests that cases of Chagas disease are likely underreported.

The presence of triatomines infected with *T. cruzi* was also evident in the communities Boca do Moa and Nova Cintra, where positive cases of Chagas disease were identified. This raises concerns, as triatomines serve as a link for the transmission of Chagas disease, both in terms of vector transmission and through contaminated food from palms—the location where triatomines were collected. This highlights the need for local health authorities to be alert and implement preventive measures against the spread of the disease. For greater contribution, more studies need to be conducted, involving a larger number of communities and participants, to bring health and knowledge to these hard-to-reach areas and to further elucidate aspects of the current Chagas disease scenario in the vast Amazon region.

## Figures and Tables

**Table 1:** Distribution of the population by residence, sex, and age group in 2019 in the municipalities of the states of Acre and Amazonas, Brazil.

Communities	N	Sex		Age Group	
		Female	Male	0 to 17 Years	18 Years or Older
Nova Cintra *	195	54% (105)	46% (90)	44% (86)	56% (109)
Boca do Moa **	69	58% (40)	42% (29)	36% (25)	64% (44)
Boca de Carapanatuba ***	47	60% (28)	40% (19)	68% (32)	32% (15)
Carará ***	83	56% (46)	44% (37)	43% (36)	57% (47)
Crioulo ***	17	65% (11)	35% (6)	53% (9)	47% (8)
Malvinas ***	08	50% (4)	50% (4)	63% (5)	37% (3)
Restauração ***	08	50% (4)	50% (4)	100% (8)	00% (0)
São Rafael ***	03	00% (0)	100% (3)	100% (3)	00% (0)
Tabuleta ***	34	59% (20)	41% (14)	41% (14)	59% (20)
Vila Torres ***	13	62% (8)	38% (5)	62% (8)	38% (5)
Total	477	56% (266)	44% (211)	47% (226)	53% (251)

Legend: N, absolute number; %, percentage. * Municipality of Rodrigues Alves, Acre, Brazil; ** Municipality of Cruzeiro do Sul, Acre, Brazil; *** Municipality of Humaitá, Amazonas, Brazil.

**Table 2:** Prevalence of Chagas disease cases by studied municipality/community in 2019 in the municipalities of the states of Acre and Amazonas, Brazil.

Municipality/Community	Sample Size			Diagnosis by *T. cruzi*	Prevalence
	Women	Men	Total		
Cruzeiro do Sul/Boca do Moa	40	29	69	1	1.44%
Rodrigues Alves/Nova Cintra	105	90	195	7	3.58%
Amazonas/Communities of Humaitá	121	92	213	0	0
Total	266	211	477	8	1.67

**Table 3:** Positive cases for Chagas disease by laboratory method in 2019 in the municipalities of the states of Acre and Amazonas, Brazil.

Positive Cases	Age	Sex	Tests Applied for Diagnosis			
			Thick Smear	ELISA	HAI	IFI
1 NC	23	F	N	P	P	N.A
2 NC	29	F	N	P	P	N.A
3 NC	16	F	N	P	P	N.A
4 NC	59	F	N	P	N	Indeterminate
5 NC	26	M	N	P	Indeterminate	P
6 NC	63	M	N	P	P	N.A
7 NC	22	M	N	P	P	N.A
1 BM	61	M	N	P	P	N.A

Legend: NC: Nova Cinta; BM: Boca do Moa; F: female; M: male; P: positive; N: negative; N.A: Not Analyzed; ELISA: Enzyme-Linked Immunosorbent Assay; HAI: Indirect Hemagglutination; IFI: Indirect Immunofluorescence.

**Table 4:** Triatomines collected in the Nova Cintra Community, Municipality of Rodrigues Alves, Acre, Brazil, 2019.

Seq	Palm	Developmental Stage	Sex	Species	*T. cruzi*
01	*Attalea butyraceae*	Adult	Female	*R. montenegrensis*	N
02	*Attalea butyraceae*	Adult	Female	*R. montenegrensis*	P
03	*Attalea butyraceae*	3rd-Instar Nymph	N.A	*Rhodnius* sp.	N
04	*Attalea butyraceae*	2nd-Instar Nymph	N.A	*Rhodnius* sp.	N
05	*Attalea butyraceae*	3rd-Instar Nymph	N.A	*Rhodnius* sp.	N
06	*Attalea butyraceae*	2nd-Instar Nymph	N.A	*Rhodnius* sp.	N
07	*Attalea butyraceae*	4th-Instar Nymph	N.A	*Rhodnius* sp.	N
08	*Attalea butyraceae*	3rd-Instar Nymph	N.A	*Rhodnius* sp.	P
Prevalence of triatomines infected with *T. cruzi*					25%

Legend: N.A, Not Analyzed; P, positive; N, negative.

**Table 5:** Triatomines collected in the Boca do Moa Community, Municipality of Cruzeiro do Sul, Acre, Brazil, 2019.

Seq	Capture Location	Developmental Stage	Sex	Species	*T. cruzi*
01	*Attalea butyraceae*	5th-Instar Nymph	N.A	*Rhodnius* sp.	P
02	*Attalea butyraceae*	3rd-Instar Nymph	N.A	*Rhodnius* sp. (standard *R. pictipes*/*R. stali*)	P
03	*Attalea butyraceae*	2nd-Instar Nymph	N.A	*Rhodnius* sp.	P
04	*Attalea butyraceae*	3rd-Instar Nymph	N.A	*Rhodnius* sp.	P
05	*Attalea butyraceae*	3rd-Instar Nymph	N.A	*Rhodnius* sp.	N
06	*Attalea butyraceae*	1st-Instar Nymph	N.A	*Rhodnius* sp.	N
07	*Attalea butyraceae*	1st-Instar Nymph	N.A	*Rhodnius* sp.	N
08	*Attalea butyraceae*	3rd-Instar Nymph	N.A	*Rhodnius* sp.	P
09	*Attalea butyraceae*	3rd-Instar Nymph	N.A	*Rhodnius* sp. (standard *R. pictipes*/*R. stali*)	P
Prevalence of triatomines infected with *T. cruzi*					66.67%

Legend: N.A, Not Analyzed; P, positive; N, negative.

## Data Availability

Data sharing is not applicable to this article, as no new data were created or analyzed in this study.
